# Eye Movement Latency Coefficient of Variation as a Predictor of Cognitive Impairment: An Eye Tracking Study of Cognitive Impairment

**DOI:** 10.3390/vision7020038

**Published:** 2023-05-01

**Authors:** Megan Polden, Trevor J. Crawford

**Affiliations:** 1 Department of Primary Care & Mental Health, University of Liverpool, Liverpool L3 5TR, UK; 2 Health Research, Lancaster University, Lancaster LA1 4YW, UK; 3 Psychology Department, Lancaster University, Lancaster LA1 4YW, UK

**Keywords:** Alzheimer’s disease, saccades, eye movements, latency, coefficient of variation

## Abstract

Studies demonstrated impairment in the control of saccadic eye movements in Alzheimer’s disease (AD) and people with mild cognitive impairment (MCI) when conducting the pro-saccade and antisaccade tasks. Research showed that changes in the pro and antisaccade latencies may be particularly sensitive to dementia and general executive functioning. These tasks show potential for diagnostic use, as they provide a rich set of potential eye tracking markers. One such marker, the coefficient of variation (CV), is so far overlooked. For biological markers to be reliable, they must be able to detect abnormalities in preclinical stages. MCI is often viewed as a predecessor to AD, with certain classifications of MCI more likely than others to progress to AD. The current study examined the potential of CV scores on pro and antisaccade tasks to distinguish participants with AD, amnestic MCI (aMCI), non-amnesiac MCI (naMCI), and older controls. The analyses revealed no significant differences in CV scores across the groups using the pro or antisaccade task. Antisaccade mean latencies were able to distinguish participants with AD and the MCI subgroups. Future research is needed on CV measures and attentional fluctuations in AD and MCI individuals to fully assess this measure’s potential to robustly distinguish clinical groups with high sensitivity and specificity.

## 1. Introduction

Eye movements are a powerful tool for assessing cognitive functioning [1,2,3,4,5]. Alzheimer’s disease is a prominent neurodegenerative disease that results in abnormalities in the control of eye movements [6,7,8]. Due to the current clinical diagnostic tests, AD often goes undiagnosed until later stages, making treatments and interventions less effective. Treatments for AD are most effective when administered in the early stages of the disease prior to neurodegeneration in the brain becoming widespread and rendering treatments ineffective [9]. Current diagnostic methods that are capable of detecting AD in the early stages are either invasive (lumbar puncture for cerebrospinal fluid sample) or expensive (neuroimaging). Eye tracking could provide an invaluable indicator for neurodegenerative disorders and impaired cognitive functioning, offering a cost-effective and non-invasive alternative [7,10,11]. Multiple eye tracking markers for impairment were not assessed or compared. The current study aims to assess potential impairment markers on pro and antisaccade tasks and their sensitivity in identifying established dementia and the preclinical stages of mild cognitive impairment.

In clinical populations and healthy adults, the antisaccade task was widely used to assess inhibitory control [12,13]. The antisaccade task requires a participant to inhibit shifting their gaze towards the displayed target and instead look towards the opposite side [14,15]. Due to a reduction in inhibitory control, disengagement of attention, and a decline in working memory and executive functioning [16], people with AD are significantly slower at performing pro and antisaccadic eye movements resulting in an increase in mean latencies [17,18,19]. In an addition to cognitive slowing, Crawford et al. [15] demonstrated higher error rates and uncorrected errors in AD on the antisaccade task that correlated with dementia severity. Apparently, top-down executive control is required to inhibit the eye gaze from shifting towards the target, and this top-down processing requires working memory resources often impaired in people with AD [20,21].

Deficits in eye tracking performance are evident when assessing antisaccades in people with AD [5]; however, this was not fully investigated in earlier, preclinical stages, such as aMCI and naMCI groups. For a biological marker to be beneficial, it must be sensitive enough to detect subtle signs of impairment in the preclinical stage. MCI is a clinical syndrome characterised by cognitive impairments that are atypical for a person’s age. MCI was traditionally classed as a distinct stage of dementia due to the deficits not being sufficiently severe to significantly impact an individual’s daily living and capabilities [22,23]. However, there is a growing case that MCI should be classed as a preclinical stage between normal cognitive health and AD [9]. There are two subgroups of MCI, amnesic MCI (aMCI) and non-amnesic MCI (naMCI) [24]. People with aMCI experience greater memory impairments than naMCI, whereas people with naMCI often have preserved memory but display other cognitive impairments, such as executive functioning deficits. People with aMCI are deemed at a greater risk of progressing to AD then naMCI [25,26]. Previous research assessing MCI subtypes in relation to eye movement performance found that eye movement paramotors, such as latencies and error rates, were able to distinguish between naMCI and aMCI [27]. Interestingly, results show aMCI participants performed more similarly in the antisaccade task to AD participants, and naMCI more similarly to healthy controls. This provided further support for the antisaccade task as a useful task to identify and monitor cognitive impairment and even be successful in distinguishing subtle differences between MCI subgroups [28].

Research to date indicates that fluctuations of eye movement latencies could serve as an additional impairment marker [18]. When programming a saccadic eye movement, there is a decisional process that takes place prior to the eye movement [29]. This decisional process is often measured as the time taken between target onset and the threshold for triggering the goal-directed saccade. The time required to initiate a saccadic eye movement relies on the resources of executive functioning and attentional processing capabilities; therefore, impairments in these operations can result in reductions in processing speed and increased latency fluctuations. Therefore, latency variability could be an indicator of attentional fluctuations when completing these tasks. Participants with attentional deficits often show a greater fluctuation of task latencies and scores [18]. This indicates less consistency and reductions in sustained attention across the course of the task, indicating attentional processing deficiencies [30]. A measure of latency variability in pro and antisaccade tasks may offer markers for further distinctions between healthy adults and people with memory impairments.

The current study investigated attentional fluctuations using a measure of relative variability termed the coefficient of variation (CV). This measure takes the ratio of the standard deviation in relation to the mean. The higher the CV, the greater the level of dispersion around the mean score. The lower the CV percentage, the more precise and the less variable the measure is. CV could be an additional biological marker for impairment, alongside other existing eye tracking makers, such as mean latencies and error rates. Yang et al. [18] assessed CV scores of prosaccade eye movements on a gap and overlap version of the task. Results show higher CV in latencies for AD participants than for healthy adults and aMCI participants. Increased variability of accuracy and speed was also abnormally higher in AD participants in both vertical and horizontal saccades [19]. This indicates the potential for CV in latencies on the prosaccade task to distinguish between AD and healthy adults. The current study expanded on this research by assessing CV in latencies in a wider range of tasks (prosaccade and antisaccade) and in a wider group of participants, with the addition of naMCI participants. The addition of the naMCI will provide information on the potential of latency CV scores to distinguish between subgroups of MCI participants, which is vital in identifying more at-risk groups for AD.

In summary, the current study investigated the potential of mean latencies, latency CV measures, and error rates as biological markers for impairment on prosaccade and antisaccade tasks. These measures will be evaluated for their potential to detect cognitive impairment, particularly in distinguishing the preclinical stages of dementia by comparing AD, aMCI, and naMCI in relation to healthy older adults.

## 2. Materials and Methods

### 2.1. Participants

The study included 65 participants with a diagnosis of dementia due to AD (mean age =74.15, SD = 7.75), 42 with aMCI (mean age = 73.71, SD = 7.42) and 47 naMCI (mean age = 69.26, SD = 6.89), and 98 older adult controls (mean age = 67.80, SD = 8.10). The AD and MCI participants were recruited from various NHS sites and memory clinics across the UK. The AD participants met the requirements for the American Psychiatric Association’s Diagnostic and Statistical Manual of Mental Disorders (DSM IV) and the National Institute of Neurological and Communicative Disorders and Stroke (NINCDS) for AD. All AD and MCI participants received a full assessment from a qualified NHS dementia specialist. The MCI participants had a formal diagnosis and met the following criteria [31]: (1) subjective reports of memory decline (reported by individual or caregiver/informant); (2) memory and/or cognitive impairment (scores on standard cognitive tests were >1.5 SDs below age norms); and (3) activities of daily living were moderately preserved. To subgroup the MCI participants into aMCI and naMCI, the free and cued selective reminding test with immediate recall (FCSR-IR) task (see below) scores were used for classification [23].

Control participants were recruited via opportunity sampling. Participants with focal cerebral lesions, history of neurological disorders, neurodegenerative disease, cerebrovascular disease, or alcoholism were excluded. Control participants who scored less than 26 on the Montreal Cognitive Assessment (MoCA) [32] were excluded from the final analysis. All participants were deemed to have the capacity to consent to participation in the study and informed consent was obtained from all subjects involved in the study. Ethical approval was granted by the Lancaster University Ethics Committee and the NHS Health Research Authority, Greater Manchester West Research Ethics Committee.

### 2.2. Cognitive Assessments

Participants completed four cognitive assessments. The Montreal Cognitive Assessment [32] assessed cognitive impairment, with a score lower than 26 being an indicator of probable dementia. The digit span assessed verbal working memory taken from the Wechsler Adult Intelligence Scale III [33] for both forwards and backwards versions of the task. Spatial memory was assessed using the spatial span task via the use of the Corsi block [33] for both forwards and backwards versions. As recommended by the International Working Group on Alzheimer’s Disease, the FCSR-IC task was conducted [34] due to its high sensitivity in differentiating between AD and MCI subgroups [35]. Participants were asked to memorise 16 drawings (presented 4 at a time), and these were linked to category cues to be used as memory prompts. Participants were asked to search the four images, and point to and name the item (for example onion) based on the category clue verbally given (a vegetable). The card was then removed, and participants asked to recall the four items based on the category clue. Participants were reminded of any items and corresponding cue if unable to recall or identify. This procedure was repeated for all 16 items. The test phase consisted of three recall trials, each preceded by a 20 s counting distractor task. For each trial, participants were given two minutes to freely recall the items. Following this, category cues were provided for the items they were unable to recall. The task provides a measure of free recall and cued recall for correct responses (a total of 48 for both scores). MCI participants who scored equal to or below 27 on the free recall score were classified as aMCI, and scores over 28 classified as naMCI, as recommended by Lemos et al. [31].

### 2.3. Eye Tracking Tasks

Eye movements were recorded via the EyeLink Desktop 1000 at 500 Hz. A chin rest was used to reduce head movements. Participants sat approximately 55 cm away from the computer monitor (60 Hz). Participant’s gazes were calibrated and validated using 9-point calibration prior to each task. The stimulus was created and controlled via the use of Experiment Builder Software Version 1.10.1630. The data were analysed and extracted using Data Viewer Software Version 3.2.

#### 2.3.1. Prosaccade Task

**Figure 1:**
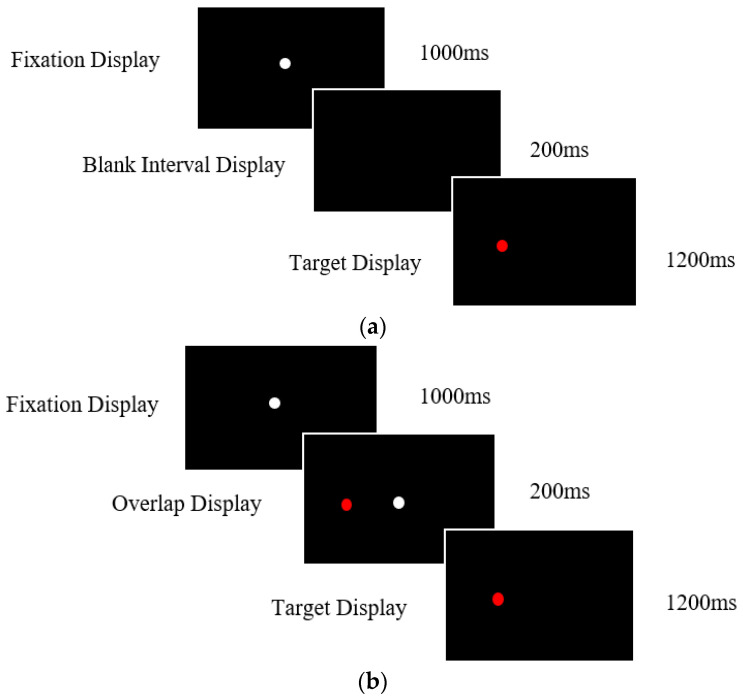
(**a**). Timings and display presentation screens for the prosaccade task gap condition. Task instructions required participants to look towards the red target, shifting their focus from the white central fixation. (**b**). Timings and display presentation screens for the prosaccade task overlap condition. Task instructions required participants to look towards the red target shifting their focus from the white central fixation.

Participants were presented with 36 gap trials followed by 12 overlap trials. A white fixation target was displayed for 1000 ms in order to centre the participants gaze, followed by a red target presented randomly to the left or right at 4° for 1200 ms. Participants were instructed to first look towards the white fixation point at the centre of the screen and then towards the red target as quickly and accurately as possible. For the gap condition, there was a blank interval screen displayed for 200 ms between the extinguishment of the white fixation target and the initial appearance of the red target. This resulted in a temporal gap in stimuli presentation (Figure 1a). In the overlap condition, the target was presented, while the central fixation point remained on the screen for 200 ms. There was an overlap in stimuli presentation, resulting in the target and the fixation point being displayed simultaneously for 200 ms (Figure 1b). After a short period, the central fixation was removed, and the target presented singularly for 1200 ms.

#### 2.3.2. Antisaccade Task

**Figure 2:**
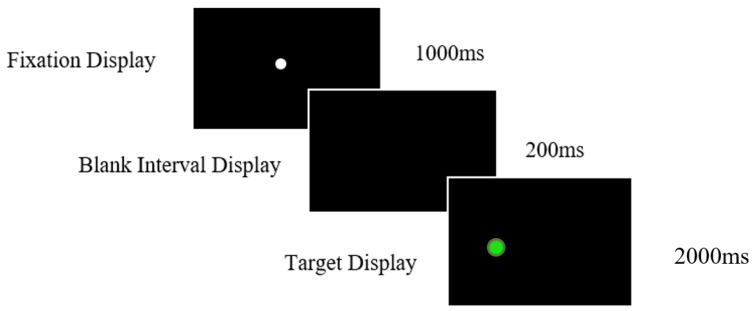
Timings and display presentation screens for the antisaccade task. Task instructions required participants to ignore the green target and move their gaze to the opposite side of the screen.

Participants completed 24 gap trials and 4 practice trials. Participants were presented with a central white fixation for 1000 ms, followed by a green target on the left or right side of the screen presented for 2000 ms. Participants were instructed to direct their gaze and attentional focus to the opposite side of the screen to which the target appeared (Figure 2). There was a 200 ms gap in the presentation of the fixation point and the target in which a blank interval screen appeared. Participants needed to generate the saccade to the opposite side of the screen of where the target was displayed to perform a successful antisaccade.

### 2.4. Data Processing

The raw data were extracted and analysed via EyeLink using DataViewer Software Version 3.2. A bespoke software [36] was then used to analyse the data offline. This software removed spikes and noise by filtering out frames with a velocity signal greater than 1500 deg/s or with an acceleration signal greater than 100,000 deg^2^/s. The EyeLink Parser was used to detect the fixations and saccadic events and the saccades were extracted alongside multiple temporal and spatial variables. Trials were removed in cases when the participant did not direct their gaze to the central fixation. The temporal window of 80–700 ms was used and measured from the onset of the target display. Anticipatory saccades made prior to 80 ms and excessively delayed saccades made after 700 ms were removed. Latency CV scores were calculated using the following formula: latency standard deviation/mean latency × 100.

### 2.5. Statistical Analysis

The results were analysed using ANOVA models via SPSS Version 28. Participant’s eye tracking mean latencies and latency standard deviations were compared with performance for the cognitive assessments and group effects were assessed. One MCI participant was excluded from the analysis due to insufficient eye tracking data. To examine the effect of participant group on cognitive performance (MoCA, digit span, spatial span, and FCSR-IC), an ANOVA was performed. For the eye tracking tasks (prosaccade gap, prosaccade overlap, and antisaccade task) ANOVA’s were performed comparing the effects of participants in the group on eye tracking mean latencies and CV scores. Pearson correlations assessed the relationship between the eye tracking markers and cognitive assessment performance.

## 3. Results

### 3.1. Cognitive Assessments

An ANOVA was performed to assess the effect of the group on cognitive performance on the MoCA, digit span, spatial span, and FCSR task. For the MoCA, results reveal a significant effect of the participant group, F (3, 247) = 73.99, *p* < 0.001. Post hoc comparisons revealed AD produced significantly lower scores compared to older adults and naMCI participants. There was no significant difference between AD and aMCI participants for MoCA score. There was a significant difference between the MCI subgroups, with naMCI producing significantly higher scores than aMCI. Further aMCI and naMCI participants also expectedly scored lower when compared to older controls (see Table 1).

For the digit span task, there was an effect of the participant group (F (3, 228) = 6.98, *p* < 0.001), with AD participants scoring lower than older controls on the task. Further aMCI also scored significantly lower than controls on the task, although no significant difference was found between controls and naMCIs. There were no further significant differences between the groups.

There was a significant group effect on spatial task performance, F (3, 222) = 15.10, *p* < 0.001. AD participants scored lower compared to control and naMCI participants. Both MCI subgroups produced significantly lower scores when compared with controls. There were no further significant differences between the MCI subgroups.

The FCSR task has a significant effect on participant group F (3, 163) = 20.96, *p* < 0.001 when assessing total task score with AD participants scoring lower than controls and both MCI subgroups. There were no significant differences between the MCI subgroups and the controls.

### 3.2. Prosaccade Task—Gap Condition

#### 3.2.1. Mean Reaction Times and Coefficient of Variation Group Effects

Results reveal no significant effects by the participant group on prosaccade mean reaction times, F (3, 169) = 1.78, *p* = 0.153 (Table 2). When assessing CV measures, there was a significant effect by the participant group on CV scores, F (3, 169) = 2.70, *p* = 0.047. Post hoc comparisons revealed that the older adult group displayed lower coefficient of variation scores indicating less variation in prosaccade reaction times during the task; however, this was not statistically significant. Interestingly, there was no significant difference between AD and older controls.

#### 3.2.2. Correlations between Prosaccade Markers and Cognitive Assessments

Correlations were conducted to compare the eye tracking measures (mean latencies and CV scores) and the cognitive assessment scores. Due to the variations between the participant groups, correlations were assessed for the groups individually. Interestingly, there was no single task that consistently correlated with mean latencies or CV across the groups. The aMCI group showed correlations between CV score and the digit span task backwards version (r (17) = −0.486, *p* = 0.048), and for the spatial span task, forwards (r (17) = −0.492, *p* = 046), backwards (r (17) = −0.512, *p* = 0.036), and total scores (r (17) = −0.548, *p* = 0.023), and also for MoCA task score (r (17) = −0.551, *p* = 0.022). Participants with higher task scores produced lower CV, indicating less variation in latencies across prosaccade trials. The aMCI group also showed a significant correlation between mean latencies and MoCA task score (r (17) = −0.543, *p* = 0.024). However, this was not consistent across the other groups. The controls showed a significant correlation between CV score and backwards digit span score (r (56) = −0.299, *p* = 0.025) and total score (r (56) = −0.268, *p* = 0.046), again with higher task score correlating with less fluctuation in latencies. Further, the AD and naMCI group did not show any correlations between eye tracking latencies and cognitive assessments, indicating a weak link between these markers.

### 3.3. Prosaccade Task—Overlap Condition

#### 3.3.1. Mean Reaction Rimes and Coefficient of Variation Group Effects

When assessing group effects on mean reaction times, Table 3 reveals there were no significant differences between the groups, F (3, 167) = 2.55, *p* = 0.058. The overlap condition often leads to a delay in disengaging attention from the fixation point, which potentially resulted in less variation between groups when initiating the saccade. Table 3 reveals no significant differences in CV scores across the participant groups (F (3, 167) = 0.354, *p* = 0.786), indicating limited potential for distinction between participant groups for this task.

#### 3.3.2. Correlations between Prosaccade Markers and Cognitive Assessments Overlap

Similar to the prosaccade gap condition, there was little consistency across groups when assessing correlations. The aMCI group showed a correlation between mean latencies and spatial span total score (r (23) = 0.454, *p* = 0.030) and FCSR free recall score (r (29) = 0.418, *p* = 0.024), but unlike the gap condition, there were no correlations between CV scores and the cognitive task score. The control group showed a significant correlation between mean latencies and the FCSR total score with participants who scored higher on the task displaying lower mean latencies (r (31) = −0.442, *p* = 0.013). There were no significant correlations found for the AD and naMCI consistent with the gap condition.

### 3.4. Antisaccade Task

#### 3.4.1. Correct Trials Mean Reaction Times and Coefficient of Variation Group Effects

Results reveal the significant effect of the participant group on antisaccade mean reaction times, F (3, 238) = 13.54, *p* < 0.001. Post hoc comparisons revealed that the AD group produced significantly slower saccade reaction times compared to healthy older adults (Table 4), indicating reductions in processing speed and inhibitory control deficits. The AD and aMCI group produced comparable saccade reaction times, supporting previous research that AD and aMCI show similar impairments and deficits. The AD and naMCI produced significantly different results, with the AD group producing slower saccade reaction times than the naMCI group. The naMCI group performed similarly to healthy controls, with no significant difference in saccade reaction times. The aMCI group produced significantly slower saccade reaction times than the naMCI group, which again supports previous research on distinctions between naMCI and aMCI participants, with aMCI performing more similarly to the AD and the naMCI more similarly to the healthy older controls (Table 4). There were no significant differences in measures of CV between the participant groups, F (3, 238) = 2.21, *p* = 0.087. This indicates that the variability of scores and performance of the antisaccade task is not affected by disease. The AD and MCI group do not display differences in CV when compared to healthy adults, indicating comparable and typical levels of attentional fluctuation on the task.

#### 3.4.2. Correlations between Antisaccade Markers and Cognitive Assessments

In contrast to the prosaccade task, the AD group revealed a significant correlation between antisaccade mean latencies and the digit span forwards score (r (60) = −0.324, *p* = 0.011). Further, the CV score correlated with FCSR total scores (r (44) = −0.389, *p* = 0.009). Participants who score higher on these cognitive tasks produced lower and less variable mean latencies. The only correlation found for the aMCI group was between the CV score and digit span forwards task score with, again, a higher task score indicating lower CV scores and less variable latencies (r (38) = −0.357, *p* = 0.028). For the naMCI, the only correlation was between the CV score and spatial span forward score (r (43) = −0.416, *p* = 0.006). The control group showed correlations between saccadic mean latencies and MoCA score (r (88) = −0.294, *p* = 0.005). These results indicate that there is not a sole cognitive task that consistently correlates with the eye tracking markers across the groups. However, it is clear from the results that higher cognitive functioning and higher task scores often lead to lower mean latencies and saccadic processing speeds and less variation in latencies, indicating less attentional fluctuation.

### 3.5. Error Rates

An error was defined as a saccade in the direction of the presented distractor target. This was determined based on the first saccade in the direction of left or right. An ANOVA was performed to assess the group effects on percentage of error trials. Results reveal the significant effect of participant groups on percentage error rate (F (3, 243) = 12.96, *p* < 0.001), as previously reported in this cohort [20]. Post hoc comparisons revealed that AD participants displayed a significantly higher number of errors compared to naMCI and controls (Table 5). AD participants produced a similar number of errors on the task as aMCI, resulting in no significant difference between AD and aMCI participants. The aMCI group produced significantly higher percentage error rates compared to naMCI and controls, indicating that they performed more similarly to the AD group than the naMCI group. Further, there was no significant difference between error rates when comparing the naMCI and the control group. This indicates that naMCI produces an error rate more similarly to controls than aMCI and AD participants. Error rates on the antisaccade task may be successful at distinguishing between AD and aMCI participants from naMCI and controls.

## 4. Discussion

The current study assessed the effectiveness CV as an additional biological marker alongside well-founded measures such as mean latencies and antisaccade error rates. The study assessed mean latencies and CV on the prosaccade and antisaccade tasks. The CV measure provides a proxi measurement of latency fluctuations throughout the task. Given the previous research finding greater attentional fluctuation (determined by higher CV scores) for prosaccade eye tracking tasks in people with MCI and AD [18,19], it was predicted that this finding would be replicated in the current study and may be evident on other similar eye tracking tasks, such as the antisaccade task. However, results from the current study show no significant differences in CV measures across the groups for the pro or antisaccade tasks. This failure to replicate could be due to the lack of sensitivity and robustness of CV scores, particularly in detecting more subtle variations between AD and MCI subgroups.

Another key finding revealed that antisaccade mean latencies were able to distinguish participants with AD from older controls and between the MCI subgroups showing high sensitivity. Participants with AD produced significantly slower mean latencies, indicating a greater difficulty in generating the saccade and a reduction in processing speed. This finding is supported by previous research showing inhibitory control impairments resulting in difficulties performing correct antisaccades, leading to speed reductions and increased difficulty in triggering saccades [37,38]. Previous research [39] demonstrated that eye movement latencies greatly rely on attentional processes, often impaired in people with AD [40]. The slowing in saccade latencies is likely the result of these attentional impairments [41]. The current study provides further support for the effectiveness of mean latencies and indicates sufficient sensitivity to distinguish between MCI subgroups and preclinical stages of AD.

It was previously demonstrated that people with AD show more variable latencies than older controls and people with MCI, which suggests that higher latency variability is related to greater attentional fluctuation [30,42]. More variable latencies on the task indicate that people with AD have less sustained attentional focus on the task compared to older controls and MCI participants, and this is likely to be due to damage to regions of the brain responsible for executive functioning and attentional processing. Yang et al. [18] found a higher latency CV, increased variability of accuracy, and abnormally high latencies for people with AD compared to healthy adults and MCI participants. It was stated that the latency and latency variability abnormalities reflect deficits of cerebral areas involved in the execution and triggering of saccades. However, the results from the current study do not support these findings, and instead show that levels of variation and CV scores were comparable across the groups. It is possible that variations in attentional fluctuation may only be evident in more advanced stages of AD; however, it is also possible that the experimental tasks and analysis methods employed in the current study are not sensitive enough to detect more subtle CV variations in early to moderate stages of AD. CV scores on other eye tracking tasks may prove more sensitive to variations in CV scores in early to moderate stages of AD and preclinical stages, and this requires further assessment in the literature. However, previous research showed higher CV scores and increased attentional fluctuation in MCI participants on the tasks used in this study, which does not support this conclusion [18]. These inconsistent findings indicate that CV may not be a reliable and robust marker for cognitive impairment as previously thought in the literature. More research is needed to assess CV scores and their robustness for distinguishing clinical and non-clinical groups in eye tracking tasks.

A further key finding was the clear distinction seen on the antisaccade task between the MCI subgroups. The aMCI group produced significantly higher antisaccade mean latencies compared to naMCI. This indicates that aMCI have greater deficits in generating and executing saccadic eye movements and the decisional process prior to an eye movement. The time required to initiate a saccade relies on executive functioning and attentional processing capabilities, and therefore impairments in these areas result in a slowing in processing speed and increased latencies. The current study indicates reduced capabilities in executive functioning and attentional processes in aMCI compared to naMCI. Antisaccade mean latencies were comparable for the AD and aMCI and significantly different from the naMCI and controls, indicating similar processing and executive functioning capabilities between aMCI and AD participants. The naMCI group performed more similarly to controls again, further emphasising this MCI distinction. People with aMCI are more likely to progress to develop AD, whereas naMCI are less likely to progress to an AD diagnosis, and the pattern of results in the current study supports this deviation. The antisaccade task appears to be a useful tool at highlighting the distinction between these MCI subgroups and provides support for the argument of MCI, particularly aMCI, to be assessed as a preliminary stage prior to AD or full-blown dementia. The clear distinctions between these groups for the antisaccade task is valuable when assessing biological markers between MCI subgroups to provide vital information on the likelihood of an individual to develop AD and as an indication of the severity of this progression.

The relationship of eye tracking mean latencies and CV with paper-based cognitive assessments was assessed. The results reveal that cognitive task scores correlated with mean latencies and CV scores; however, the specific cognitive assessment correlating with the eye tracking measure varied for each participant group. The overall tread showed that higher scores on the cognitive assessments correlated with faster mean latencies and lower CV scores. This finding adhered with previous research findings that cognitive ability is reflected in pro-saccade and antisaccade eye movement performance [43,44]. However, these results also indicate that different cognitive tasks are more effective in predicting mean latencies and CV depending on the participant’s group. This brings into question the robustness of eye tracking measurement in directly predicting cognitive ability, as mean latencies and CV score only correlate with certain cognitive assessments, which vary depending on participant group and ability. Further, it must also be considered that the cognitive assessments are not sensitive enough to correlate with more subtle variations and changes in mean latencies and CV scores across the groups. This should be assessed with a wider battery of cognitive assessments to further assess consistency between groups.

In summary, the current study assessed the disease effect on pro and antisaccade eye movement latencies, CV, and error rates. Certain parameters on the antisaccade task are capable of distinguishing between AD participants, MCI subgroups, and older control participants, but it is clear that research into the effectiveness of CV as a biological marker for impairment is further required, as results do not provide clear evidence of increase in attentional fluction in AD and MCI participants. This conflicts with previous findings, which showed promising findings for CV as an additional biological marker; however, more research is required to fully assess the robustness and full potential of this variable.

## Figures and Tables

**Table 1:** Table displaying means, standard deviations, and post hoc contrasts for MoCA, digit span, spatial span, and FCRS task score for all participant groups.

	Alzheimer’s Disease (*n* = 65)		aMCI (*n* = 42)		naMCI (*n* = 46)		Healthy Older Controls (*n* = 98)				Post Hoc Contracts (*p* Values)			
											Disease Effects			
	M	SD	M	SD	M	SD	M	SD	AD vs. OC	AD vs. aMCI	AD vs. naMCI	aMCI vs. naMCI	aMCI vs. OC	naMCI vs. OC
MoCA	19.98	5.71	20.93	4.46	25.34	2.17	28.02	1.79	<0.001 *	0.577	<0.001 *	<0.001 *	<0.001 *	<0.001 *
Digit Span	15.64	4.12	16.35	3.66	16.66	4.79	18.72	4.48	<0.001 *	0.850	0.631	0.988	0.023 *	0.050
Spatial Span	11.34	3.12	12.58	3.10	13.00	2.55	14.56	2.81	<0.001 *	0.178	0.022 *	0.919	0.004 *	0.021 *
FCSR-IC	36.48	14.72	45.10	4.41	47.39	1.29	47.73	0.94	<0.001 *	<0.001 *	<0.001 *	0.592	0.401	0.996

Note. Dependent variable: task score. * Significant at *p* < 0.05 level.

**Table 2:** Table displaying means and standard deviations for mean latencies and CV scores and post hoc contracts for the prosaccade task gap condition.

	Alzheimer’s Disease (*n* = 31)		aMCI (*n* = 29)		naMCI (*n* = 27)		Healthy Older Controls (*n* = 71)				Post Hoc Contracts (*p* Values)			
											Disease Effects			
	M	SD	M	SD	M	SD	M	SD	AD vs. OC	AD vs. aMCI	AD vs. naMCI	aMCI vs. naMCI	aMCI vs. OC	naMCI vs. OC
Mean Latencies	215	31.88	201	39.14	226	60.33	203	48.56	0.648	0.770	0.826	0.351	0.997	0.163
Coefficient of Variation	23.14	10.03	26.93	17.09	25.57	15.62	19.77	12.41	0.627	0.687	0.916	0.720	0.060	0.271

Note. Dependent variable: Reaction times.

**Table 3:** Table displaying means and standard deviations for mean latencies and CV scores and post hoc contracts for the prosaccade task overlap condition.

	Alzheimer’s Disease (*n* = 43)		aMCI (*n* = 29)		naMCI (*n* = 27)		Healthy Older Controls (*n* = 69)				Post Hoc Contracts (*p* Values)			
											Disease Effects			
	M	SD	M	SD	M	SD	M	SD	AD vs. OC	AD vs. aMCI	AD vs. naMCI	aMCI vs. naMCI	aMCI vs. OC	naMCI vs. OC
Mean Latencies	274	57.61	234	62.45	273	74.51	254	71.51	0.462	0.070	0.999	0.127	0.509	0.601
Coefficient of Variation	37.94	19.29	38.96	18.20	36.44	19.04	34.93	18.15	0.857	0.997	0.989	0.966	0.814	0.986

Note. Dependent variable: reaction times.

**Table 4:** Table displaying means and standard deviations for mean latencies and CV scores and post hoc contracts.

	Alzheimer’s Disease (*n* = 65)		aMCI (*n* = 42)		naMCI (*n* = 47)		Healthy Older Controls (*n* = 88)				Post Hoc Contracts (*p* Values)			
									Disease Effects					
	M	SD	M	SD	M	SD	M	SD	AD vs. OC	AD vs. aMCI	AD vs. naMCI	aMCI VS naMCI	aMCI vs. OC	OC naMCI VS
Mean Latencies	404.34	86.34	418.91	81.70	363.05	61.61	338.12	83.91	<0.001 *	.804	0.041 *	0.008 *	<0.001 *	0.320
Coefficient of Variation	23.57	10.43	20.55	5.80	25.04	6.79	24.74	10.30	0.858	0.376	0.854	0.133	0.080	0.998

Note. Dependent variable: reaction times. * Significant at *p* < 0.05 level

**Table 5:** Table displaying mean and standard deviations and post hoc contracts for percentage error rates for all participant groups.

	Alzheimer’s Disease		aMCI		naMCI		Healthy Older Controls				Post Hoc Contracts (*p* Values)			
								Disease Effects						
	M	SD	M	SD	M	SD	M	SD	AD vs. OC	AD vs. aMCI	AD vs. naMCI	aMCI VS naMCI	aMCI vs. OC	OC naMCI VS
Percentage error rate	26.13	28.80	30.11	30.02	12.40	10.75	10.36	10.98	<0.001 *	0.773	0.004 *	0.001 *	<0.001 *	0.951

Note. Dependent variable: percentage error rate. * Significant at *p* < 0.05 level

## Data Availability

Data are available via open science framework and can be accessed using the following link https://osf.io/n2mxa/ URL (accessed on 30 April 2023) DOI 10.17605/OSF.IO/N2MXA.
